# Successful switch from enzyme replacement therapy to miglustat in an adult patient with type 1 Gaucher disease: a case report

**DOI:** 10.1186/s13256-016-1060-y

**Published:** 2016-11-08

**Authors:** Gaetano Giuffrida, Rita Lombardo, Ernesto Di Francesco, Laura Parrinello, Francesco Di Raimondo, Agata Fiumara

**Affiliations:** 1 Regional Reference Center for Rare Diseases, Clinical Division of Hematology and Transplantation, PO Ferrarotto Hospital, Azienda Ospedaliera-Universitaria Policlinico-Vittorio Emanuele, Via Citelli, 6-95100, Catania, Italy; 2 Department of Clinical Medicine and Pediatrics, Pediatric Clinic, Gaspare Rodolico Azienda Ospedaliera-Universitaria Policlinico-Vittorio Emanuele, Catania, Italy

**Keywords:** Gaucher disease, Enzyme replacement therapy, Substrate reduction therapy, Miglustat

## Abstract

**Background:**

Gaucher disease is one of the most common lipid-storage disorders, affecting approximately 1 in 75,000 births. Enzyme replacement therapy with recombinant glucocerebrosidase is currently considered the first-line treatment choice for patients with symptomatic Gaucher disease type 1. Oral substrate reduction therapy is generally considered a second-line treatment option for adult patients with mild to moderate Gaucher disease type 1 who are unable or unwilling to receive lifelong intravenous enzyme infusions. The efficacy and safety of the oral substrate reduction therapy miglustat (Zavesca®) in patients with Gaucher disease type 1 have been established in both short-term clinical trials and long-term, open-label extension studies. Published data indicate that miglustat can be used as maintenance therapy in patients with stable Gaucher disease type 1 switched from previous enzyme replacement therapy.

**Case presentation:**

We report a case of a 44-year-old Caucasian man with Gaucher disease type 1 who was initially treated with enzyme replacement therapy but, owing to repeated cutaneous allergic reactions, had to be switched to miglustat after several attempts with enzyme replacement therapy. Despite many attempts, desensitization treatment did not result in improved toleration of imiglucerase infusions, and the patient became unwilling to continue with any intravenous enzyme replacement therapy. He subsequently agreed to switch to oral substrate reduction therapy with miglustat 100 mg twice daily titrated up to 100 mg three times daily over a short period. Long-term miglustat treatment maintained both hemoglobin and platelet levels within acceptable ranges over 8 years. The patient’s spleen volume decreased, his plasma chitotriosidase levels stayed at reduced levels, and his bone mineral density findings have remained stable throughout follow-up. The patient’s quality of life has remained satisfactory. Miglustat showed good gastrointestinal tolerability in this patient, and no adverse events have been reported.

**Conclusions:**

Oral miglustat therapy proved to be a valid alternative treatment to intravenous enzyme replacement therapy for long-term maintenance in this patient with Gaucher disease type 1, who showed persistent allergic intolerance to imiglucerase infusions. This report exemplifies the type of patient with Gaucher disease type 1 who can benefit from switching from enzyme replacement therapy to substrate reduction therapy.

## Background

Gaucher disease (GD) is an autosomal recessive inherited lysosomal storage disease caused by a deficiency of the enzyme β-glucocerebrosidase (also called *β-glucosidase* [GBA]), which catalyzes the degradation of the sphingolipid glucocerebroside (glucosylceramide) into ceramide and glucose [1–3]. This deficiency, caused by mutations on chromosome 1q21 [4], leads to the accumulation of glucocerebroside in the lysosomes of macrophages that collect in the liver, spleen, bone marrow, and skeleton [5]. Subsequent progressive visceral involvement has a large impact on both quality of life and life expectancy.

Traditionally, on the basis of the presence or absence of neurological symptoms and signs, GD is categorized into three subtypes [6, 7]. The most common form, Gaucher disease type 1 (GD1), is generally considered to be nonneuronopathic in nature and is characterized by hepatosplenomegaly, hematological abnormalities (anemia and thrombocytopenia), and skeletal manifestations such as osteopenia, fragility fractures, and bone pain. Primarily neuronopathic forms are less common. GD type 2 is an acute neuronopathic variant associated with early-onset and rapidly progressive neurological deterioration as well as visceral manifestations. GD type 3 is a chronic neuronopathic form characterized by later onset and more protracted neurological and visceral involvement. However, a number of published reports indicate that a significant proportion of patients with GD1 have neurological abnormalities [8–10]. The different types of GD are therefore perhaps better viewed as occurring across a phenotypic continuum [11, 12]. In light of the weight of published evidence, it seems appropriate to drop the terms *type 2* and *type 3 GD* and more representative to apply the terms *acute neuronopathic* and *chronic neuronopathic GD* [13].

Enzyme replacement therapy (ERT) with recombinant glucocerebrosidase is currently considered the first-line treatment choice for patients with symptomatic GD1, providing proven beneficial effects on hematological and visceral manifestations and improvements in some skeletal indices [14, 15]. Oral substrate reduction therapy (SRT) is generally considered a second-line treatment option for adult patients with mild to moderate GD1 who are unable or unwilling to receive lifelong intravenous enzyme infusions [16].

The iminosugar miglustat (*N*-butyldeoxynojirimycin, Zavesca®; Actelion Pharmaceuticals, Allschwil, Switzerland) is an inhibitor of glucosylceramide synthase (ceramide-specific glucosyltransferase), which is the enzyme that catalyzes the first committed step in the biosynthesis of glycosphingolipids [17]. The efficacy and safety of miglustat in patients with GD1 have been established in both short-term clinical trials and long-term, open-label extension studies [16, 18, 19]. Further studies have indicated that miglustat can be used as a maintenance therapy in patients with stable GD1 switched from previous ERT [20].

We report a case of a man with GD1 who was initially treated with ERT but, owing to repeated cutaneous allergic reactions, had to be switched to miglustat after several attempts with ERT. The patient’s clinical details are discussed in relation to the safety profile of miglustat.

## Case presentation

A 44-year-old Caucasian man was admitted to our department in March 2002 with thrombocytopenia and leukocytosis. The same signs had appeared 2 years before in another hospital, without a definitive diagnosis. At admission, he had a platelet count of 123,000/mm^3^ and a white blood cell count of 8750/mm^3^. Physical and neurological examinations did not reveal any physical symptoms.

The patient’s clinical history revealed a spontaneous right lung collapse in 1987 (treated by drainage for 1 week) and an abdominal cardiac valve defect with gastroesophageal reflux since 1989. Liver and spleen hemangioma were detected by abdominal ultrasound. A bone marrow biopsy showed 70 % cellularity, with approximately half of the cell population comprising well-differentiated histiocytes. The cell cytoplasm was distended and closely packed with fine granules staining blue with a May-Grünwald-Giemsa preparation, and centrally located nuclei in some cells were displaced toward the periphery in others. These features were consistent with sea-blue histiocytes.

During follow-up, the patient’s initial hemoglobin (Hb) concentration and platelet count were 15.4 g/dl and 154,000/mm^3^, respectively, and his plasma chitotriosidase activity was 2150 nmol/h/ml. In May 2002, a fluorometric assay showed a β-glucosidase activity of 0.30 nmol/h/mg (range 1.7–4.3 nmol/h/mg), which is compatible with GD. This diagnosis was confirmed by molecular genetic testing, showing homozygous N370S mutations. At this point, the patient’s spleen volume (calculated on the basis of polar diameter) was 12.5 cm^3^. In addition, bone mineral density (BMD) measurement by dual-energy X-ray absorptiometry revealed severe osteoporosis (T-score greater than −2.5).

In April 2003, several months after admission, ERT with imiglucerase (Cerezyme®; Genzyme, Cambridge, MA, USA) was started with a dosage of 60 IU/kg of body weight every other week. Approximately 1 year later (March 2004), the patient underwent upper-right lobectomy with mediastinal lymphadenectomy for treatment of lung cancer (clear-cell adenocarcinoma). In follow-up approximately 2.5 years later (December 2005), a clear improvement in BMD (T-score −1 and less than or equal to −2.5) (Table 1) and a substantial (> 70 %) decrease in plasma chitotriosidase to 616 nmol/h/ml were observed. The patient’s standard hematological parameters remained stable (Hb 15.4 g/dl, platelet count 126,000/mm^3^). In July 2004, a cutaneous allergic reaction appeared on the patient’s abdomen and shoulder during the ninth infusion of imiglucerase, which led to stoppage of the infusion and subsequent treatment with methylprednisolone 20 mg. Similar adverse reactions occurred at the 10th and 11^th^ infusions. Thereafter the patient was prepared 30 minutes before starting ERT with intravenous chlorpheniramine maleate 5 mg to prevent allergic manifestations. No further infusion reactions occurred up to the 20^th^ administration, at which point the patient developed urticarial, pruritus, diarrhea, and chest discomfort.

**Table 1:** Dual-energy X-ray absorptiometry findings

Date	T-score	Treatment
November 2002	Greater than −2.5	ERT
December 2003	−1/-2.5	ERT
July 2005	−1/−2.5	ERT
May 2006	−1/−2.5	ERT
May 2008	−1/−2.5	SRT
March 2011	−1.5	SRT
April 2012	−1.5	SRT

*ERT* Enzyme replacement therapy, *SRT* Substrate reduction therapy

**Fig. 1:**
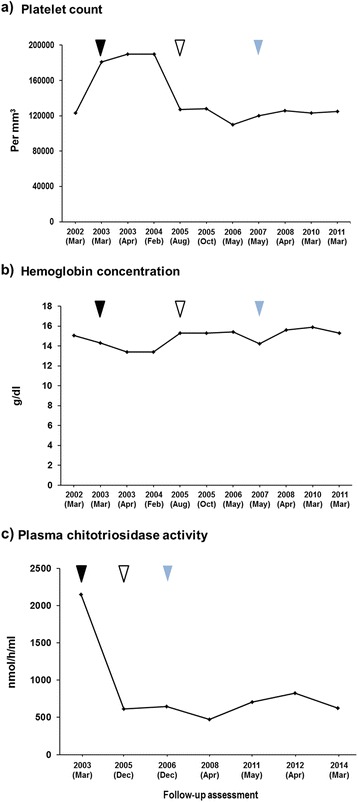
Hematological parameters during initial enzyme replacement therapy and subsequent miglustat treatment. *Black*, *white*, and *blue triangles* represent start of enzyme replacement therapy, cessation of enzyme replacement therapy, and start of miglustat therapy. **a**) platelet count during treatment: *Black triangle* represent start of enzyme replacement therapy, *white triangle* represent cessation of enzyme replacement therapy and *Blue triangle* represent start of miglustat therapy. **b**) hemoglobin concentration during treatment: *black triangle* represent start of enzyme replacement therapy, *white triangle* represent cessation of enzyme replacement therapy and *blue triangle* represent start of miglustat therapy. **c**) plasma chitotriosidase activity: *Black triangle* represent start of enzyme replacement therapy, *white triangle* represent cessation of enzyme replacement therapy and *blue triangle* represent start of miglustat therapy

The patient became unwilling to continue with any intravenous ERT because of his recurrent allergic manifestations, which were poorly prevented by antihistamine therapy. However, he later agreed to switch to oral therapy with miglustat (Zavesca®, Actelion Pharmaceuticals), which was started at a dosage of 100 mg twice daily and titrated up to 100 mg three times daily over a short period according to the manufacturer’s instructions [21]. He has since remained on miglustat therapy with good compliance and has not reported any adverse events. His core hematological parameters and further BMD findings have remained stable throughout follow-up (Fig. 1a–c). His spleen volume decreased from 12.0 cm^3^ at miglustat initiation to 11.1 cm^3^ in December 2015, after a total of 12 years of ERT and SRT. The patient has never had hepatosplenomegaly, and his liver and renal parameters were always normal before, during, and after ERT. To date, no bisphosphonate therapy for osteoporosis has ever been applied. Last follow-up was in December 2015.

## Discussion

GD is one of the most common lipid-storage disorders, affecting approximately 1 in 75,000 births [22]. The homozygous N370S mutation on the chromosome 1q21 is the most common genotype observed in affected patients. Our patient, who was homozygous for N370S, was treated in accordance with current treatment recommendations and other literature reports.

The standard of care for the treatment of the systemic, nonneuronopathic manifestations of GD is ERT with a recombinant analogue of human GBA. Intravenous ERT infusions for GD have been shown to improve hematological parameters (increased Hb concentrations and platelet counts), reduce organomegaly, and decrease bone involvement [14, 15]. In most cases, the therapeutic response to ERT reaches a plateau within the first 2–5 years [14, 15].

The response of our patient to ERT was initially good, including improvements in Hb and platelets, decreased bone involvement, and reduced chitotriosidase levels that were in line with findings reported in the literature [14, 15]. However, repeated infusion reactions curtailed the continued use of ERT in this case. The development of immunoglobulin G antibodies in response to intravenous infusions has been reported in approximately 15 % of ERT-treated patients, with many of those affected experiencing a range of immune reactions, including pruritus, flushing, tachycardia, and hypotension [23–26]. These side effects are usually mild [26, 27], allowing continuation of therapy after slowing of infusion rates or the use of pharmacological support with corticosteroids and antihistamines. In patients who respond poorly to these treatments, a desensitization protocol has successfully been implemented in both pediatric and adult patients [28, 29]. Unfortunately, despite many attempts, premedication with methylprednisolone and chlorpheniramine maleate did not result in improved toleration of imiglucerase infusions in our patient. ERT was therefore stopped with the patient’s consent, and it was decided that miglustat therapy should be commenced.

Miglustat acts as a competitive inhibitor of glucosylceramide synthase, thereby decreasing the synthesis and accumulation of glucosylceramide and related glycosphingolipids in reticuloendothelial cell lysosomes [30]. The efficacy of miglustat has previously been demonstrated over the short term and long term in patients with GD regarding both reduced visceral involvement (sustained reductions in both liver and spleen volumes) and improved hematological parameters [16, 18, 19, 31, 32]. Data also indicate that miglustat is effective as maintenance therapy in patients with stable GD switched from previous ERT [33–35]. In particular, this agent has shown beneficial effects on BMD indices in cortical and trabecular bone, bone marrow burden scores, and bone pain, which are possibly related to its physicochemical properties that enable a wide distribution throughout body tissues [32, 36, 37]. Published data derived from a long-term safety surveillance program for miglustat have demonstrated its safety in patients with GD treated for up to 9 years [38, 39].

Overall, the long-term effects of miglustat in this case are in agreement with previous findings. Both Hb and platelet count remained within acceptable ranges throughout 8 years of follow-up on miglustat since the patient was switched from previous ERT. His spleen volume decreased, and his plasma chitotriosidase levels were maintained at reduced levels. The patient’s quality of life has remained satisfactory.

It is well known on the basis of early clinical trials in patients with GD that miglustat can lead to gastrointestinal disturbances, mainly during the initial weeks or months of treatment [40]. This is due to reversible inhibition by orally administered miglustat of intestinal disaccharidases, including sucrose and maltase, and to a lesser extent lactase [40, 41]. As a result of this activity, the amount of undigested oligosaccharides in the gastrointestinal lumen increases, which leads to osmotic diarrhea and related symptoms [40]. However, in recent years, the management of these side effects in miglustat-treated patients has improved. In particular, simple dietary modifications such as restriction of dietary disaccharide intake, ideally commenced before initiation of treatment, has proved useful in reducing gastrointestinal side effects and in maintaining body-weight gain within normal limits in pediatric patients [40, 42, 43]. Notably, probiotics rich in *Saccharomyces boulardii*, combined with miglustat dose escalation, have been reported to confer further possible benefits in this respect [44]. We suggested restriction of disaccharides for our patient, and we observed acceptable gastrointestinal tolerability based on the patient’s self-reports.

## Conclusions

We report a case of a patient with GD who underwent long-term SRT treatment with miglustat. He showed improvements in visceral and hematological parameters during initial ERT, but he had to stop ERT because of an unacceptable level of adverse infusion reactions. After he was switched to miglustat, his core disease parameters, particularly bone indices, remained stable or improved, and his quality of life was maintained. In terms of tolerability, dietary modifications and general patient education regarding gastrointestinal disturbances that can occur during initial miglustat treatment appeared to result in acceptable compliance with treatment. While reports of previous open-label trials have indicated that miglustat can, in general, act as a maintenance therapy in patients switched from previous intravenous treatment with ERT, this case provides a specific example of the type of patient with GD1 in whom switching to oral SRT can be of particular benefit.

## Abbreviations

BMD: Bone mineral density
ERT: Enzyme replacement therapy
GBA: β-glucocerebrosidase
GD: Gaucher disease
GD1: Gaucher disease type 1
Hb: Hemoglobin
SRT: Substrate reduction therapy
